# Long-term anti-tumor effects following both conventional radiotherapy and FLASH in fully immunocompetent animals with glioblastoma

**DOI:** 10.1038/s41598-022-16612-6

**Published:** 2022-07-19

**Authors:** Emma Liljedahl, Elise Konradsson, Emma Gustafsson, Karolina Förnvik Jonsson, Jill K. Olofsson, Crister Ceberg, Henrietta Nittby Redebrandt

**Affiliations:** 1 The Rausing Laboratory, Division of Neurosurgery, Department of Clinical Sciences, Lund University, Lund, Sweden; 2 Medical Radiation Physics, Department of Clinical Sciences, Lund University, Lund, Sweden; 3 Department for Geosciences and Natural Resource Management, University of Copenhagen, Copenhagen, Denmark; 4 Department of Neurosurgery, Skåne University Hospital, Rausing Laboratory, Lund University, BMC D10, 221 84 Lund, Sweden

**Keywords:** Neurology, Oncology

## Abstract

Radiotherapy can induce an immunological response. One limiting factor is side effects on normal tissue. Using FLASH radiotherapy, side effects could possibly be reduced. The efficacy of FLASH in relation to conventional radiotherapy (CONV-RT) has not been extensively explored in fully immunocompetent animals. Fully immunocompetent Fischer 344 rats were inoculated with NS1 glioblastoma cells subcutaneously or intracranially. Radiotherapy was delivered with FLASH or CONV-RT at 8 Gy × 2 (subcutaneous tumors) and 12.5 Gy × 2 (intracranial tumors). Cured animals were re-challenged in order to explore long-term anti-tumor immunity. Serum analytes and gene expression were explored. The majority of animals with subcutaneous tumors were cured when treated with FLASH or CONV-RT at 8 Gy × 2. Cured animals could reject tumor re-challenge. TIMP-1 in serum was reduced in animals treated with FLASH 8 Gy × 2 compared to control animals. Animals with intracranial tumors survived longer when treated with FLASH or CONV-RT at 12.5 Gy × 2, but cure was not reached. CONV-RT and FLASH were equally effective in fully immunocompetent animals with glioblastoma. Radiotherapy was highly efficient in the subcutaneous setting, leading to cure and long-term immunity in the majority of the animals.

## Introduction

There are few effective treatment options for patients with glioblastoma, and the survival is poor, with median survival only around 12–15 months in well selected patients included in clinical trials with conventional therapy^1^. The majority of the patients would not even meet the inclusion criteria of recent clinical phase III trials^2^. According to guidelines it is recommended that patients with recurrent or progressing glioblastomas are included in clinical studies, but also adult patients with newly diagnosed glioblastomas should be considered^1^.

Radiotherapy is one of the cornerstones in oncological treatment of glioblastoma^1^. A limiting factor concerning radiotherapy is the side effects, not least neuro-cognitive decline, in the setting of intracranial tumors. With FLASH radiotherapy, irradiation is delivered at an ultra-high dose rate, compared to conventional radiotherapy (CONV-RT)^3^. There is some evidence supporting that severe toxicities could be reduced with FLASH compared to CONV-RT^4^, whereas there is less evidence supporting a maintained anti-tumor effect^3,4^. Hopefully, FLASH radiotherapy could constitute a possible way of achieving equivalent anti-tumor effects, but with reduction of normal tissue damage^5^. However, demonstrating that equal anti-tumor effects are actually achieved, has not been fully analyzed^3^, and even less so in fully immunocompetent preclinical models^5,6^. Regarding experimental glioblastoma, delayed growth could be demonstrated in nude mice with xenotransplanted tumors with sparing of cognitive side effects after FLASH versus CONV-RT with some but not all of the irradiation protocols analyzed^5^.

According to previous research by our research group^7^ and others^8^, radiotherapy delivered at optimal doses and fractions can induce an effective immune response, which seems to function in synergy with immunotherapy, also in the intracerebral setting. However, delivery of radiotherapy with many fractions decreases lymphocyte count in circulating blood^9^. In a consecutive study of patients with grade III astrocytoma, glioblastoma or oligodendroglioma (according to previous WHO classification system), corticosteroids and radiotherapy (mean dose 59 Gy during 43 days) led to severe immunosuppression^10^. Furthermore, in patients treated according to the Stupp protocol^11^ with radiotherapy at 60 Gy over 30 fractions and concomitant and adjuvant Temozolamide, a long-lasting immunosuppression could be observed, with low CD4 counts lasting throughout one year of follow-up^12^. Exposure of the large circulating blood flow through the brain could be the cause of radiation-induced lymphopenia^9^. Decreasing the fractions to five or less, and increasing the dose in each fraction, on the other hand, could lead to reduced lymphopenia according to simulations^9^. We have modulated optimal radiation dose and fractionation in relation to immunotherapy^13^. In order to achieve maximal synergetic effect with the specific immunotherapy studied within that protocol (IDO inhibition), the numbers of fractions should be decreased to between two to six fractions^13^. In relation to the abscopal effect, with tumor regression outside the field of irradiation, immunotherapy and irradiation seems to yield a good response with few fractions (three to five) but relatively large doses/fractions (6–8 Gy) also in other models^14^.

In the present study we explored if long-lasting anti-tumor response could be achieved using CONV-RT or FLASH in a fully immunocompetent rat glioblastoma model, which has been lacking in studies covering FLASH radiotherapy. We explored irradiation efficacy both on subcutaneous and intracranial tumors. An advantage with rats is that they are considered to be physiologically more similar to humans compared to for example mice^15^. In previous studies, models with immune deficient animals have been mainly used, and long-term anti-tumor effects have not been fully explored^5^. We used fully immunocompetent rats with a newly developed glioblastoma tumor cell line, generating tumors with an infiltrative growth pattern and perivascular dissemination, hard to cure in an experimental setting^7,16^. We chose doses of irradiation which are supposed to yield an immune response, at least when combined with immunotherapy^7,14,17^, with irradiation at 8 Gy × 2 in the subcutaneous model. We increased the doses in animals with intracranial tumors to 12.5 Gy × 2, since previous data from our tumor model showed that CONV-RT at 8 Gy × 2 combined with immunotherapy did not result in cure in the majority of animals with intracranial tumors^7^.

### The specific aims were to explore

1. If there was a difference in anti-tumor efficacy following radiotherapy with CONV-RT or FLASH in fully immunocompetent animals irradiated with low total doses in an early phase of tumor growth, with 8 Gy × 2 for subcutaneous tumors, and 12.5 Gy × 2 for intracranial tumors.
2. If irradiation with CONV-RT or FLASH could generate long-term antitumor effects in fully immunocompetent animals.
3. The effect of the local tumor microenvironment, comparing effects in subcutaneous tumors to those seen in the intracranial setting.

## Results

### In vitro data

#### In vitro colony forming assay

**Figure 1:**
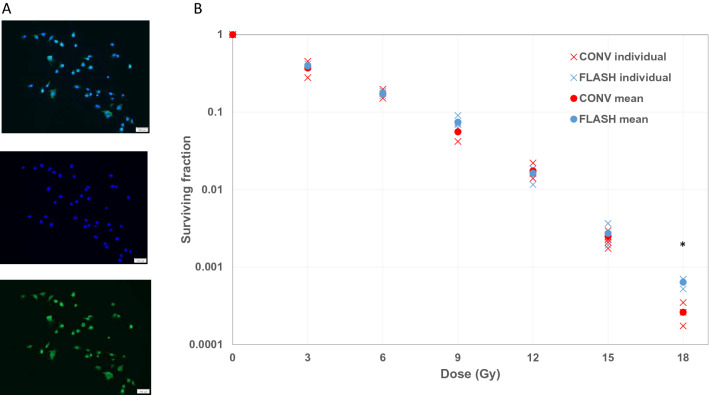
NS1 Glioblastoma cells and in vitro response to irradiation. (**A**) The NS1 rat glioma cell line used in the present study. Tumor cells are marked with dapi for nuclear staining (blue). GFP signal stands for the autofluorescence (green). (**B**) In vitro data exploring colony forming assay in relation to increased doses of CONV-RT or FLASH. Doses in the range 0–21 Gy were used for each modality. * indicates significant differences between CONV-RT and FLASH. At 21 Gy, no colonies were detected.

NS1 autofluorescence could be demonstrated as expected (Fig. 1A). The colony forming assay showed that the surviving fraction of NS1 cells depended on dose level and dose rate (Fig. 1B). At 18 Gy, there was a significantly increased survival in cells irradiated with FLASH, as compared to CONV-RT (Mann–Whitney U-test, *p* < 0.05). At a dose of 21 Gy no colonies with ≥ 50 cells were detected.

### Subcutaneous tumors

#### Significant anti-tumor effect of both FLASH and CONV-RT versus control in fully immunocompetent animals with subcutaneous glioblastoma

**Figure 2:**
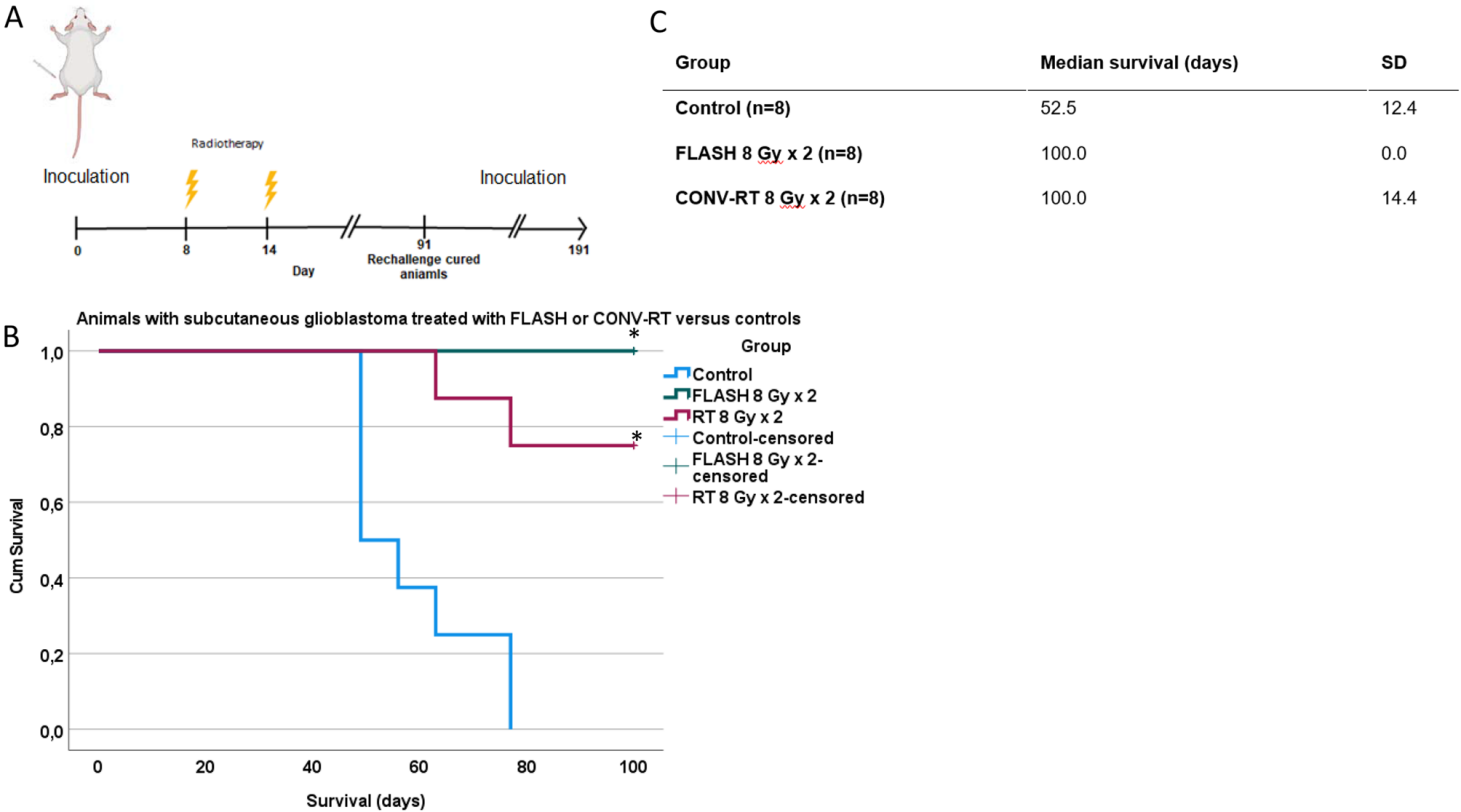
Survival in animals with subcutaneous tumors. (**A**) Experimental setup in animals with subcutaneous glioblastoma. Created with BioRender.com. (**B**) Survival (days) after first tumor cell inoculation. Survival differed significantly between the two irradiated groups compared to control animals. (**C**) Median survival in animals treated with CONV-RT 8 Gy × 2, FLASH 8 Gy × 2 or control animals.

In order to evaluate the efficacy of CONV-RT and FLASH in fully immunocompetent animals, rats were subcutaneously inoculated with NS1 tumors cells day 0. Animals were divided into groups with CONV-RT at 8 Gy × 2; FLASH at 8 Gy × 2 or unirradiated controls (n = 8/group). Control animals were inoculated with NS1 cells without any further treatment. Irradiation was delivered 8 and 14 days after inoculation (Fig. 2A). Tumor size was measured weekly and animals were euthanatized when the tumor diameter exceeded 30 mm or the tumor penetrated the skin resulting in open wounds.

Survival data was not normally distributed according to Shapiro–Wilk’s test and visual inspection of normality plots. All animals that were euthanatized had tumor size > 30 mm in diameter and all control animals developed tumors. Survival was significantly increased in the irradiated animals compared to control animals (Kruskal–Wallis *p* < 0.001; Mann–Whitney U-test with Bonferroni post-hoc adjustment CONV-RT versus control *p* = 0.004; FLASH versus control *p* < 0.001; FLASH versus CONV-RT *p* = 1.0) (Fig. 2B,C). There was no significant difference in survival between FLASH versus CONV-RT.

**Figure 3:**
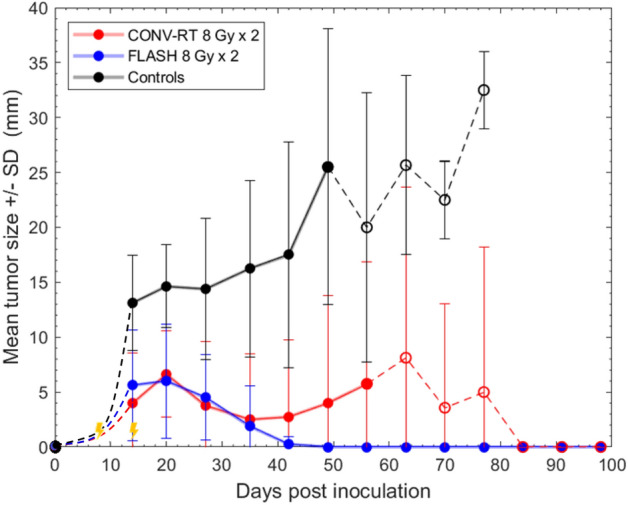
Tumor size in animals with subcutaneous tumors. Mean tumor size ± SEM in animals with subcutaneous glioblastoma irradiated with FLASH 8 Gy × 2 or CONV-RT 8 Gy × 2, as well as non-irradiated control animals (n = 8 per group). At day 0, tumor cells were inoculated, and tumor size was set to 0 mm. During the first measurements, ≤ 7 days after inoculations, tumors were small and difficult to measure exactly, and estimated size is indicated by dotted lines (- - -). From day 14 after tumor cell inoculations, tumors were distinctly measurable, and size is indicated by dots. Lines connect the different measurements as long as all animals are represented in the individual groups, but are replaced with dotted lines (- - - -) when at least one of the animals have been euthanized due to tumor growth > 30 mm. This means that the measurement points only represent tumor size of animals still alive. Days of irradiations are marked with yellow arrows.

Next, tumor size was evaluated. Until day 49 after tumor cell inoculations, data was available from all animals (on day 49 the first control animal was euthanized due to tumor exceeding 30 mm) (Fig. 3). Data was not normally distributed as tested with Shapiro Wilk’s test (*p* < 0.05) and visual inspection of normality plots. Measurements were compared between groups as long as all animals were still alive in all groups, which was until day 49 after tumor cell inoculations. Tumor size differed significantly between control animals and those treated with FLASH 8 Gy × 2 or CONV-RT 8 Gy × 2, at measurements 14–49 days after tumor cell inoculations. Significant differences were observed already after 14 days (Kruskal–Wallis *p* = 0.004, Mann–Whitney U-test with Bonferroni adjusted post-hoc test *p* = 0.006 comparing control animals to RT 8 Gy × 2; *p* = 0.033 comparing control animals to FLASH 8 Gy × 2; *p* = 1.0 comparing RT 8 Gy × 2 to FLASH 8 Gy × 2). Differences in tumor size remained throughout the observation period, as seen after 20 days (Kruskal–Wallis *p* = 0.003, Mann–Whitney U-test with Bonferroni adjusted post-hoc test *p* = 0.018 comparing control animals to RT 8 Gy × 2; p = 0.006 comparing control animals to FLASH 8 Gy × 2; *p* = 1.0 comparing RT 8 Gy × 2 to FLASH 8 Gy × 2). After 49 days, the first control animals had to be euthanized due to large tumors. At the last measurement point with all animals still alive, 49 days after tumor cell inoculations, differences were still detected (Kruskal–Wallis *p* < 0.001, Mann–Whitney U-test with Bonferroni adjusted post-hoc test p < 0.001 comparing control animals to RT 8 Gy × 2; *p* = 0.004 comparing control animals to FLASH 8 Gy × 2; *p* = 1.0 comparing RT 8 Gy × 2 to FLASH 8 Gy × 2). There was no significant difference between animals irradiated with CONV-RT or FLASH at any of the time points between day 0 to 49 after tumor cell inoculations.

#### Long-term anti-tumor effects in cured animals with subcutaneous glioblastoma

**Figure 4:**
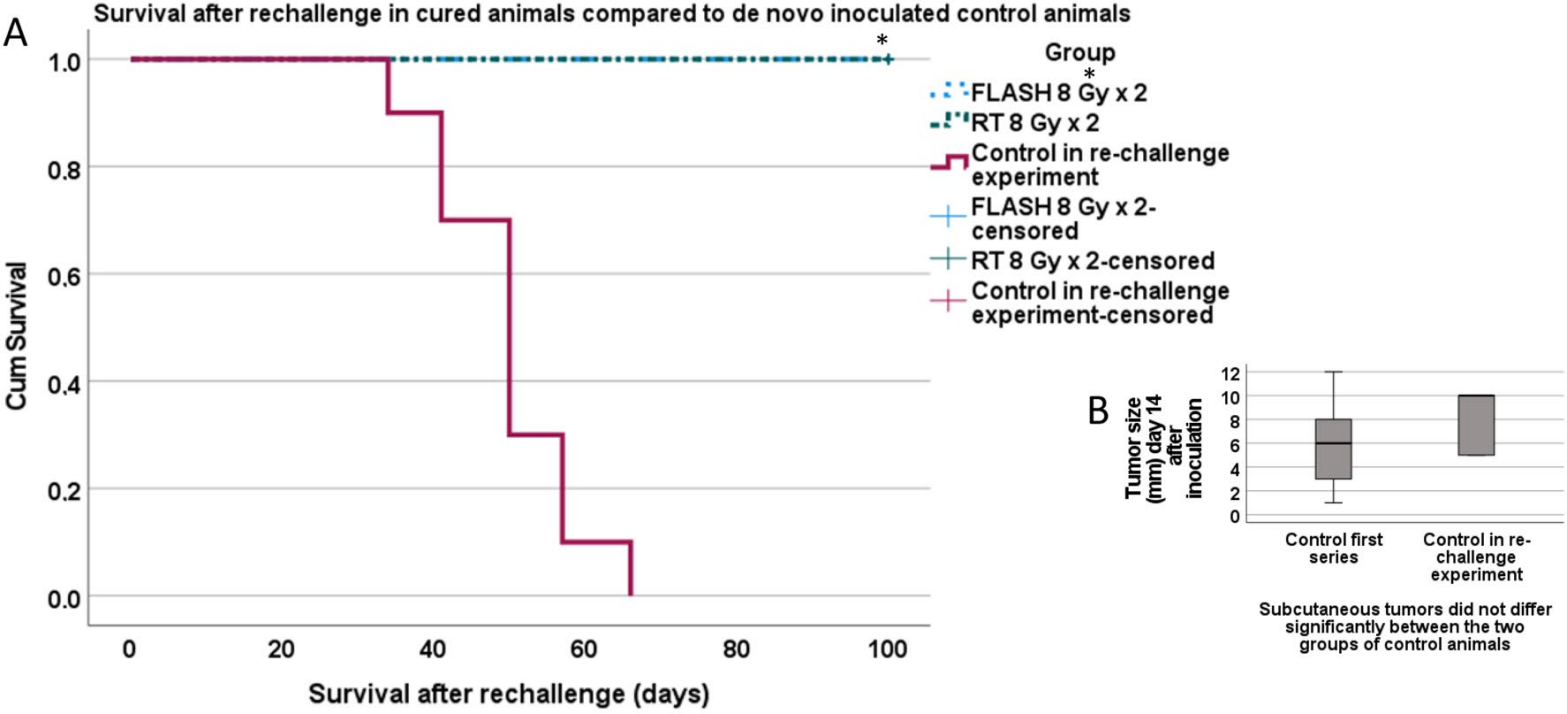
Re-challenge of animals with long-term tumor control. (**A**) All animals that managed to reject the subcutaneous tumors were subject to tumor re-challenge. They were monitored until day 191 after the first tumor cell inoculation. (**B**) Tumor size did not differ significantly between the two groups of control animals, from the first series versus the de novo inoculated controls in the re-challenge series.

At day 91 after tumor cell inoculation, 14 animals did not exhibit any sign of tumor growth and were considered to be cured; 8 treated with FLASH 8 Gy × 2 and 6 with CONV-RT 8 Gy × 2. All control animals had been euthanatized due to tumor diameter > 30 mm prior to this. The cured animals were inoculated with 50,000 NS1 cells on their contralateral flank in a re-challenge experiment. Another 10 animals were de novo inoculated control animals, not previously included in the study. All de novo inoculated control animals developed tumors. In the previously cured animals, no tumor growth could be detected during the observation period of additionally 100 days. Survival was significantly increased in all the cured animals as compared to the de novo inoculated controls (Log Rank Mantel-Cox *p* < 0.05) (Fig. 4A). At 14 days after inoculation, there was no statistically significant difference in tumor size (Mann–Whitney *p* > 0.05) in the de novo inoculated controls in the re-challenge series compared to the controls in the first series, indicating a stable tumor growth in control animals (Fig. 4B).

#### Serum analytes from animals treated with CONV-RT, FLASH and controls

Serum was collected from cured animals who had survived in total 191 days from the first tumor cell inoculation, that is 100 days from the re-challenge, and compared to that of control animals which had died due to large tumors as described above. In some cases, serum could not be analyzed due to insufficient samples. Serum levels of GM-CSF, ICAM-1, IL-2, IL-6, IL-18, TIMP-1, TNF-alpha and VEGF were compared between animals.

**Figure 5:**
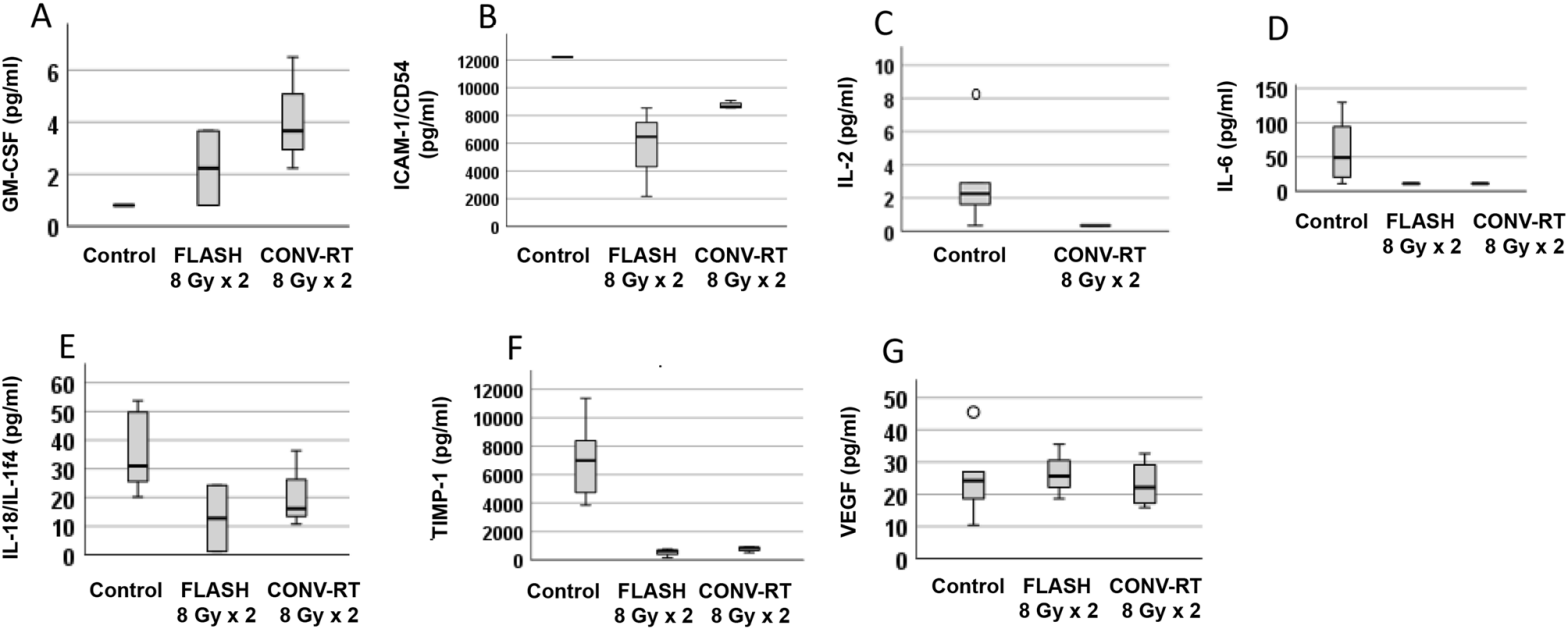
Serum levels in animals with subcutaneous tumors, comparing control animals to those irradiated with CONV-RT or FLASH. Serum was collected from the animals upon euthanasia, and those irradiated with FLASH 8 Gy × 2 and CONV-RT 8 Gy × 2 were compared to control animals. Missing data was due to values below the levels that could be technically detected, in all cases except ICAM-1/CD54 expression, where values were out of range above the levels that could be technically detected. TNF-alpha was below the reference interval in all animals, and could not be analyzed further. Non-parametric tests were used. * indicates significant differences compared to the control group. ^o^ indicates an outlier. (**A**) GM-CSF in n = 3 control animals, n = 2 FLASH 8 Gy × 2 and n = 3 CONV-RT 8 Gy × 2 treated animals. GM-CSF in a healthy animal was out of range below detectable levels. No significant difference was observed between different groups. (**B**) ICAM-1/CD54 in n = 1 control, n = 3 FLASH 8 Gy × 2 and n = 4 CONV-RT 8 Gy × 2 treated animals. ICAM-1/CD54 in healthy animal = out of range above the reference interval. N = 6 control animals exhibited values above the reference interval. No significant difference was observed amongst animals with detectable levels of ICAM-1/CD54. (**C**) IL-2 in n = 6 control versus n = 1 CONV-RT 8 Gy × 3 treated animals. IL-2 in healthy animal = 2.91 pg/ml. No significant difference was observed. (**D**) IL-6 in n = 7 control versus n = 1 FLASH 8 Gy × 2 and n = 2 RT 8 Gy × 2 treated animals. IL-6 in healthy animal = 29.77 pg/ml. No significant difference was observed. (**E**) IL-18 in n = 7 control versus n = 2 FLASH 8 Gy × 2 and n = 3 CONV-RT 8 Gy × 2 treated animals. IL-18 in healthy animal = 107.14 pg/ml. No significant difference was observed. (**F**) TIMP-1 in n = 7 control versus n = 3 FLASH 8 Gy × 2 and n = 4 CONV-RT 8 Gy × 2 treated animals. TIMP-1 in healthy animal = 1498.14 pg/ml. The levels differed significantly between controls and FLASH irradiated animals (Kruskal–Wallis *p* = 0.006, Mann–Whitney U-test with post-hoc Bonferroni correction *p* = 0.012 regarding FLASH 8 Gy × 2 versus control; *p* = 0.066 regarding CONV-RT 8 Gy × 2 versus control and *p* = 1.0 regarding FLASH 8 Gy × 2 versus CONV-RT 8 Gy × 2). (**G**) VEGF in n = 7 control versus n = 3 FLASH 8 Gy × 2 and n = 4 CONV-RT 8 Gy × 2 treated animals. VEGF in healthy animal = 24.2 pg/ml. No significant difference was observed.

One healthy animal without any previous tumor was tested too, and its serum levels are described in the figure legend in relation to the individual measurements (Fig. 5A–G). If serum expression was below or above technically detectable levels, it was reported as out of range. Comparing long-term survivors that had been treated with FLASH 8 G × 2 or CONV-RT 8 Gy × 2 demonstrated that there was no difference between these groups of animals regarding any of the serum analytes. However, TIMP-1 levels, were significantly reduced in animals that had been treated with FLASH 8 Gy × 2 compared to control animals (Fig. 5F), wheras those treated with CONV-RT 8 Gy × 2 did not reach statistical significance. The remaining serum analytes did not differ between irradiated animals with FLASH 8 Gy × 2 or CONV-RT 8 Gy × 2 compared to control animals.

### Intracranial tumors

#### Increased survival but no cure in animals with intracranial glioblastoma irradiated with FLASH 12.5 Gy × 2 or CONV-RT 12.5 Gy × 2

**Figure 6:**
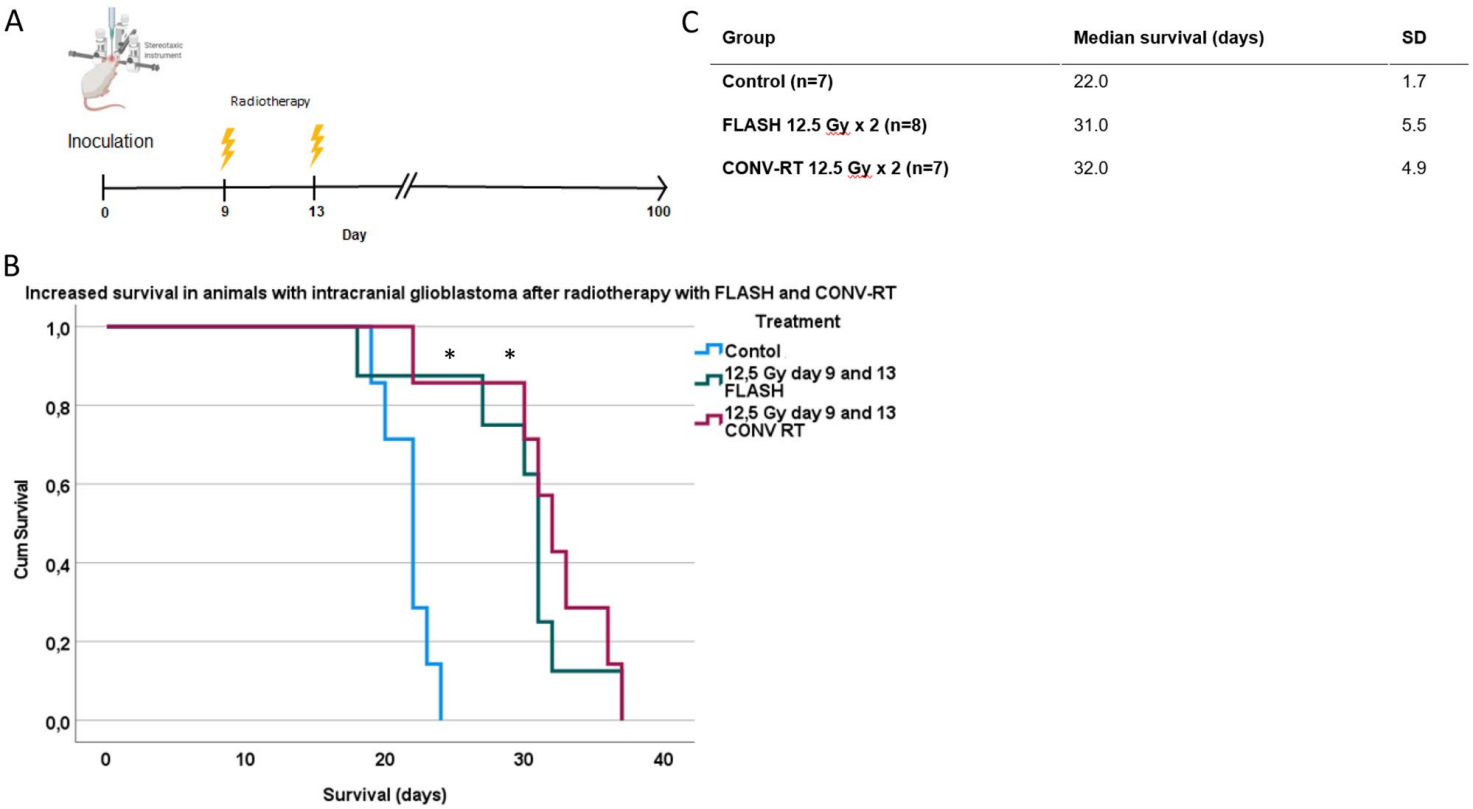
Survival in animals with intracranial tumors. (**A**) Experimental setup in animals with intracranial glioblastoma. Created with BioRender.com. (**B**) Survival was significantly increased in animals irradiated with CONV-RT or FLASH compared to control animals. There was no significant difference between the two irradiation modalities in relation to survival. (**C**) Survival data.

In order to evaluate the effect of CONV-RT versus FLASH in the intracranial setting, animals were inoculated intracranially with glioblastoma tumor cells. 7 animals were control animals, 8 were treated with FLASH 12.5 Gy × 2 and 7 were treated with CONV-RT 12.5 Gy × 2, 9 and 13 days after tumor cell inoculations (Fig. 6A). Animals were monitored on a daily basis and euthanized if they met the criteria defined by the ethics regulations, including impaired general condition and neurological deficits. Survival data was normally distributed (Shapiro–Wilk’s test and visual inspection of normality plots).

Survival was significantly increased in the irradiated animals versus control animals (Log Rank Mantel-Cox p = 0.002 comparing FLASH to control and *p* = 0.001 comparing CONV-RT to control). There was no difference between the two irradiation modalities (*p* > 0.05) (Fig. 6B,C).

#### Serum analytes from animals treated with CONV-RT, FLASH and control animals

**Figure 7:**
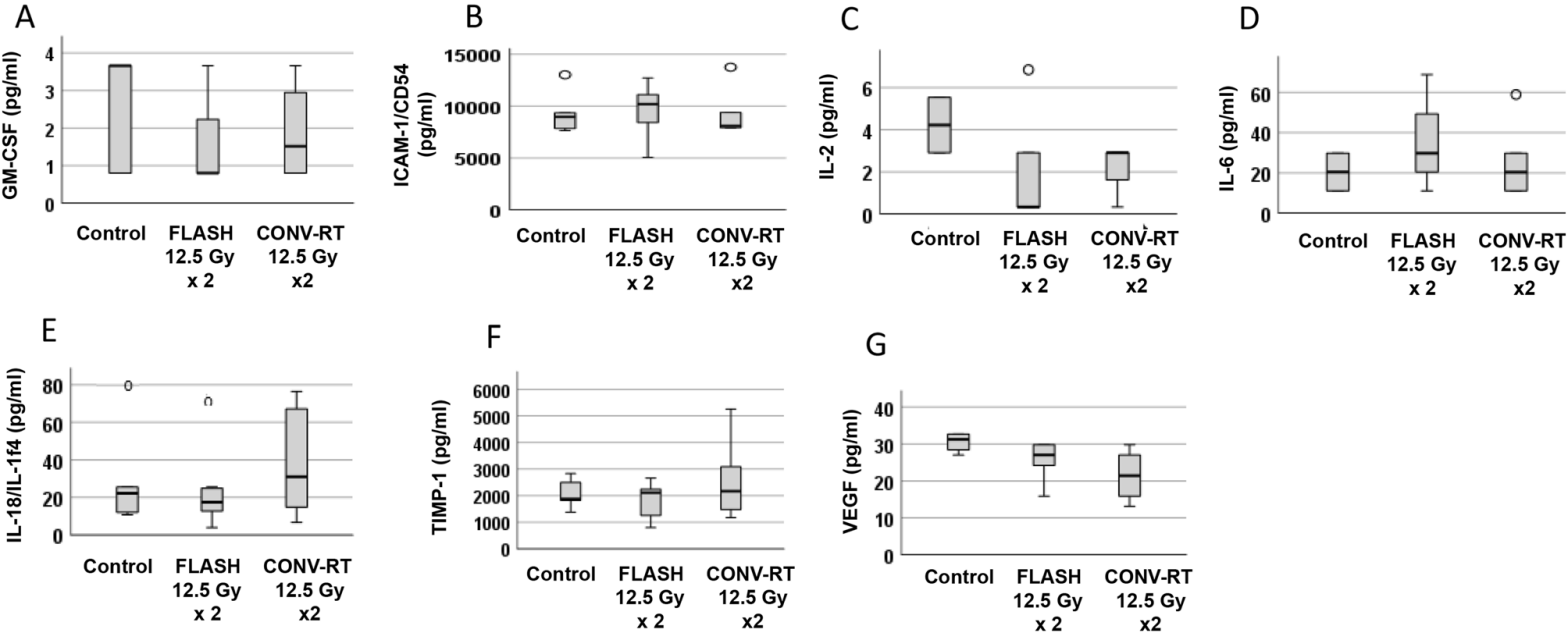
Serum levels in animals with intracranial tumors treated with FLASH, CONV-RT or control animals. Serum was collected upon euthanasia and compared between animals that had received FALSH-RT, CONV-RT or were control animals. Missing data was due to values below those that could be technically detected. TNF-alpha was out of range below the reference interval in all animals except one, and thus could not be analyzed further. * indicates significant differences compared to the control group. ^o^ indicates an outlier. (**A**) GM-CSF in n = 5 control versus n = 4 FLASH 12.5 Gy × 2 and n = 4 CONV-RT 12.5 Gy × 2 treated animals. GM-CSF in healthy animal was out of range below detectable levels. No significant difference was observed. (**B**) ICAM-1/CD54 in n = 5 control versus n = 6 FLASH 12.5 Gy × 2 and n = 5 CONV-RT 12.5 Gy × 2 treated animals. GM-CSF in healthy animal was out of range below detectable levels. No significant difference was observed. (**C**) IL-2 in n = 2 control versus n = 5 FLASH 12.5 Gy × 2 and n = 3 CONV-RT 12.5 Gy × 2 treated animals. IL-2 in healthy animal = 2.91 pg/ml. No significant difference was observed. (**D**) IL-6 in n = 2 control versus n = 3 FLASH 12.5 Gy × 2 and n = 5 CONV-RT 12.5 Gy × 2 treated animals. IL-6 in healthy animal = 29.77 pg/ml. No significant difference was observed. (**E**) IL-18 in n = 6 control versus n = 7 FLASH 12.5 Gy × 2 and n = 5 CONV-RT 12.5 Gy × 2 treated animals. IL-18 in healthy animal = 107.14 pg/ml. No significant difference was observed. (**F**) TIMP-1 in n = 6 control versus n = 7 FLASH 12.5 Gy × 2 and n = 5 CONV-RT 12.5 Gy × 2 treated animals. TIMP-1 in healthy animal = 1498.14 pg/ml. No significant difference was observed. (**G**) VEGF in n = 6 control versus n = 7 FLASH 12.5 Gy × 2 and n = 5 CONV-RT 12.5 Gy × 2 treated animals. VEGF in healthy animal = 24.2 pg/ml. The levels differed significantly between groups (Kruskal–Wallis *p* = 0.038, but no group passed Mann–Whitney U-test with post-hoc Bonferroni correction).

Serum was collected upon euthanasia in all animals. Missing values were due to expressions below the range of what could be technically detected. Whilst VEGF levels differed significantly between groups, post-hoc adjusted comparisons did not reveal any significant difference between groups (Fig. 7F). Regarding the other serum analytes, no significant differences could be seen neither between control animals or those irradiated with CONV-RT 12.5 Gy × 2 or FLASH 12.5 Gy × 2, nor between irradiated groups (Fig. 7A–G).

#### Gene expression

As presented above, glioblastomas could be cured when treated in the subcutaneous setting with radiotherapy, with long term anti-tumor immunity. On the other hand, intracranial tumors could not be cured, even though the dose was increased. In order to define the different tumor properties in the intracranial versus subcutaneous setting, gene expression was compared between control animals with tumors in these different locations.

Only tumor samples with sufficient RNA quality could be used. Preliminary analyses revealed that two subcutaneous control samples had a vastly different expression profiles from the other subcutaneous control samples and these samples were therefore excluded from all analyses. These outliers had a predominance of muscle related gene expression, which is consistent with some surrounding muscle tissue included in the tumor sample upon dissection from the subcutaneous space. In the final analysis 1 intracranial control tumor was compared to 5 subcutaneous control tumors.

**Figure 8:**
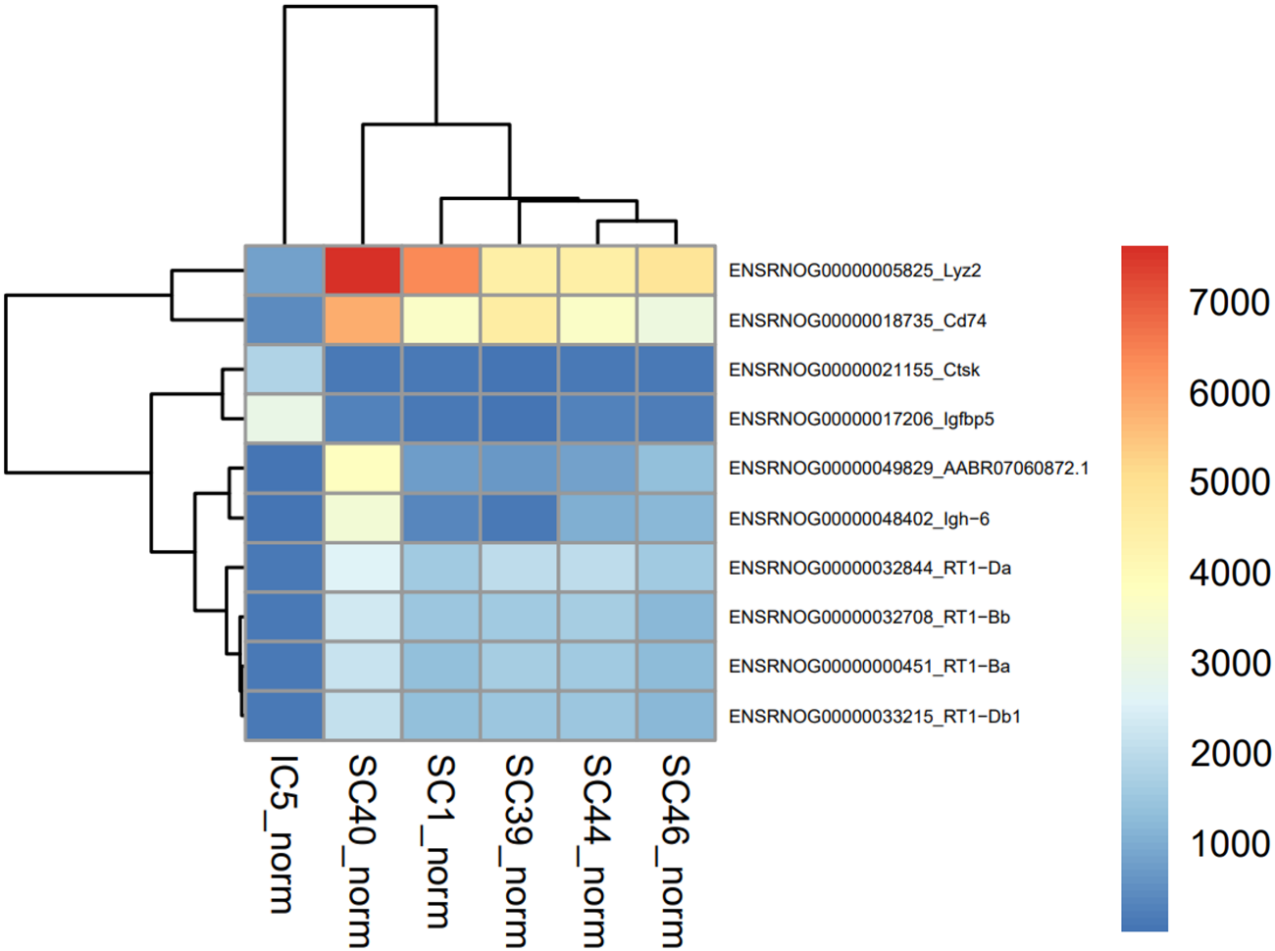
Gene expression in subcutaneous versus intracranial animals. Differently expressed genes comparing intracranial to subcutaneous control animals, with > 1000 counts per million in all samples. Abbreviations: SC = subcutaneous tumor; IC = intracranial tumor; norm = normalized to reads per million.

There was a total number of 219 differentially expressed genes between the intracranial and subcutaneous control tumors, out of a total of 32,883 included genes in the array. The 20 genes with highest fold change comparing the intracranial to the subcutaneous controls are presented in Table 1. Interestingly, the majority of these genes are associated with immune response. CD74 was among the differentially expressed genes with highest counts (> 1000 counts per million in all animals, fold change 3.44, *p* = 2.11E−09) (Fig. 8).

**Table 1:** Genes differently expressed in intracranial versus subcutaneous control animals.

Gene name	Fold change	*P* value	Function
ENSRNOG00000047414_AABR07058479.1	6.88	4.04E−08	
ENSRNOG00000022298_Cxcl11	7.08	1.41E−13	C-X-C motif chemokine
ENSRNOG00000000562_Prf1	7.09	1.51E−08	Perforin-1
ENSRNOG00000006319_Cxcr6	7.14	1.28E−06	C-X-C chemokine receptor type 6
ENSRNOG00000017749_Nkg7	7.40	9.23E−13	Natural killer cell granule protein 7
ENSRNOG00000015994_Cd3d	7.76	3.31E−12	T-cell surface glycoprotein CD3 delta chain
ENSRNOG00000022242_Cxcl9	8.01	2.08E−25	C-X-C motif chemokine
ENSRNOG00000054828_AABR07051708.1	8.20	1.27E−03	Ig-like domain-containing protein
ENSRNOG00000049829_AABR07060872.1	8.26	6.59E−10	Ig-like domain-containing protein
ENSRNOG00000003666_Jchain	8.29	5.88E−08	Immunoglobulin joining chain
ENSRNOG00000038957_RGD1305184	8.78	6.79E−08	Interferon-gamma-inducible GTPase Ifgga4 protein
ENSRNOG00000050000_AABR07034739.1	9.22	3.03E−19	Ig lambda-2 chain C region
ENSRNOG00000055193_AABR07051652.1	9.51	2.25E−06	Ig-like domain-containing protein
ENSRNOG00000009919_Acod1	9.99	3.28E−10	Aconitate decarboxylase 1
ENSRNOG00000058460_AABR07051551.2	11.44	1.30E−32	Ig-like domain-containing protein
ENSRNOG00000057165_AABR07034730.3	11.55	9.51E−04	Ig-like domain-containing protein
ENSRNOG00000050118_AABR07065750.2	11.59	2.73E−06	RCG21066
ENSRNOG00000047571_RGD1563231	12.00	2.72E−04	Similar to immunoglobulin kappa-chain VK-1
ENSRNOG00000050898_AABR07034730.2	12.04	4.86E−05	Ig-like domain-containing protein
ENSRNOG00000050708_AABR07065823.3	12.87	1.18E−06	Ig-like domain-containing protein
ENSRNOG00000048402_Igh-6	13.39	2.00E−08	Ig gamma-2B chain C region

All of the most differently expressed genes were upregulated in subcutaneous controls versus intracranial control animals.

## Discussion

In the present study, we could demonstrate a long-lasting anti-tumor effect in animals with subcutaneous glioblastoma using both CONV-RT and FLASH. Animals who had achieved long-term tumor control with no detectable tumor after 91 days, managed to reject tumor cells inoculated on their contralateral side, indicating a long-term antitumor effect. There was no difference between animals treated with FLASH or CONV-RT, indicating equal anti-tumor efficacy. In vitro, a dose–response could be demonstrated with both FLASH and CONV-RT, with a reduction of colony formation in relation to increased doses of both modalities. At irradiation doses of 3–15 Gy no difference could be detected between CONV-RT or FLASH, but at 18 Gy CONV-RT was more efficient compared to FLASH. By increasing the doses further, no colonies could be detected anymore.

Irradiation of intracranial tumors with doses at 8 Gy × 2 was avoided, since this has not resulted in long-term tumor control on a group level even in combination with immunotherapy^7^. Thus, we increased the dose to 12.5 Gy × 2 in the intracranial model. Both CONV-RT and FLASH radiotherapy prolonged survival in animals with intracranial tumors compared to control animals, but cure could not be achieved, despite an increased dose compared to the subcutaneous setting and comparable and early initiation of therapy. Irradiation was delivered to intracranial tumors 9 and 13 days after tumor cell inoculations. By the initiation of irradiation on day 9 after tumor cell inoculations, the tumor is small and previous imaging studies on the NS1 tumor with MRI revealed no detectable tumor using T2 images with contrast enhancement 7 days after intracranial inoculations, and only a very small tumor on day 10^16^.

Radiotherapy is widely used in order to achieve local tumor control. However, there is also a distant anti-tumor effect seen at non-irradiated sites, the abscopal effect. This effect seems to be immune-mediated, possibly T-cell dependent^18^. The increased understanding of the immune mediated effects of radiotherapy has led to the integration of immunotherapy with radiotherapy^7,8,13,19^. The ability to cure animals with radiotherapy and achieve long-term anti-tumor effects as presented here, further strengthens the immunological role of radiotherapy. Both animals treated with FLASH and CONV-RT demonstrated the same ability to reject tumor cells in a re-challenge experiment of cured animals with subcutaneous tumors. In serum from animals treated with FLASH at 8 Gy × 2 against subcutaneous tumors, with long-term tumor control, TIMP-1 was significantly lower compared to control animals. Interestingly, in patients with glioblastoma, it has been demonstrated that low tumor TIMP-1 immunohistochemical expression is associated with significantly longer survival, compared to high TIMP-1 expression^20^. It has been suggested that TIMP-1 has many roles, including stimulation of cell growth, inhibition of apoptosis and promotion of tumor invasion^20^.

Glioblastomas are radioresistant tumors, and even though radiotherapy is widely used against the tumors in clinical practice, development of adaptive radioresistance is a challenge^21^. Several factors contribute to this radioresistance, one of them is the tumor microenvironment, but also factors such as hypoxia, glioma stem cells, tumor heterogeneity and metabolic alterations have been suggested to contribute^21^. The constitution of the tumor microenvironment contributes to radioresistance, where glioblastoma cells can be radiosensitive in vitro, but not in vivo in xenograft models^21^. In the present study, we could demonstrate that there is a difference related to the exact location of the tumor, with intracranial tumors being much more radioresistant compared to the same tumor cells inoculated subcutaneously. All animals with intracranial tumors, although receiving higher dosages and an early intervention, had succumbed before day 40 after tumor cell inoculations, as compared to the animals with subcutaneous tumors, that showed clear reduction of tumor growth by the same time. Comparing genes from intracranial control tumor to those expressed in subcutaneous control tumors, immune related genes were frequently among the most upregulated genes in the subcutaneous tumors. CD74 belonged to the differently expressed genes with highest counts (> 1000 counts per million in all animals). CD74 is the cell membrane receptor of cytokine macrophage migration inhibitory factor (MIF)^22^. It has been demonstrated that CD74 is more expressed in gliomas compared to normal brain tissue, and is associated with immune checkpoints and inflammatory cytokines^23^. Furthermore, expression of CD74 was higher in high grade glioma compared to low grade glioma^23^.

One likely explanation of the major difference in survival between our intracranial versus subcutaneous model, is the blood–brain barrier (BBB). The BBB is a physical and chemical barrier that hinders free passage of different substances into the healthy brain^24^. In glioblastoma, this barrier is partly disrupted, but there is still a tumor burden residing behind an intact BBB^24^. Radiotherapy can affect the permeability of BBB, as seen in experimental animals with a single dose of > 10–15 Gy^25^. Early changes of BBB permeability have been demonstrated hours to days after radiotherapy, depending upon substance and animal model. However, the relationship is not fully clear yet between BBB opening and radiotherapy, and at certain time sequences, both increased and decreased permeability have been demonstrated^25^. Understanding the pattern of BBB disruption in relation to radiotherapy could improve the timing of additional therapies, not least trying to boost the immune response against the tumor. Increased disruption of the BBB, for example using focused ultrasound, could be an interesting adjunct to radiotherapy to boost BBB opening when the effect of radiotherapy has subsided in order to achieve a stronger immune mediated anti-tumor response.

### Study limitations

This study has some limitations. The major aim was to establish anti-tumor efficacy of CONV-RT and FLASH in fully immunocompetent rats with glioblastoma, and animals had to be euthanized when they had certain defined symptoms of tumor growth, in accordance with ethical guidelines. This meant that all animals could not be followed for the same amount of days. It also meant that many of the animals with subcutaneous tumors that had been irradiated actually displayed no signs of tumors at the end of the study period, yielding no material for immunohistochemical tumor analysis. However, this was also necessary, in order to explore survival effects. Some serum material could not be analyzed, due to insufficient amount or insufficient levels in relations to upper or lower bounds that are used to detect the analytes of interest. Regarding gene expression analysis, it would have been interesting to include more samples. Still, gene expression data indicated that the tumor microenvironment affects the gene expression found upon tumor tissue dissection, also when the same glioblastoma cell line was used. Further exploration of the tumor microenvironment in the intracranial versus subcutaneous setting could be developed with a larger material.

Long-term tumor control and re-challenge data could be established, which was the primary aim of this study, and it was very interesting that animals could reject a tumor re-challenge. Further mechanistic explorations would be of interest, which could be done by designing an experiment where material from all animals is analyzed at the same day post inoculations and treatment. This, however makes a survival and long-term anti-tumor response impossible to study simultaneously, and would fit within the scope of a future study.

## Conclusions

In the present study, we could demonstrate equal efficacy of CONV-RT and FLASH in fully immunocompetent animals with glioblastoma. Whereas the radiotherapy was highly efficient in the subcutaneous setting, leading to cure in the majority of the animals, the radio-resistance was pronounced in the intracranial setting. In cured animals, a long-term anti-tumor effect could be demonstrated.

## Materials and methods

### Ethics statement

This study was approved by the Animal Ethics Committee in Malmö/Lund, Sweden (permit ID 5.8.18-02383/2020 and 5.8.18-04987/2021). All experiments were performed in accordance with relevant guidelines and regulations. All efforts were made to minimize animal suffering and in accordance to ARRIVE guidelines.

### Rat glioblastoma cells

The rat glioblastoma cell line NS1^16^ was used. NS1 is a GFP (green fluorescent protein) positive tumor cell line (Fig. 1A) which can be syngeneically inoculated into Fischer 344 rats where it generates infiltrative tumors.

Sandwich Elisa was used to rule out Mycoplasma infection in the cells and supernatant and was used according to the manufacturer’s instructions (MycoProbe R&D Systems).

The rat glioblastoma cells (NS1) were cultured using RPMI-1640 (Sigma-Aldrich) medium with addition of 1% ml Na-pyruvate, 1% ml HEPES (4-(2-hydroxyethyl)-1-piperazineethanesulfonic acid), 0.1% ml gentamycin, as well as 10% inactivated fetal calf serum (heated to 56 °C for 30 min).

### In vitro colony forming assay

In vitro survival of irradiated cells was evaluated using colony forming assay. Cells were plated in T12.5 flasks (Thermo-Fisher Scientific, Waltham, MA) in a total volume of 2.5 ml, and allowed to adhere overnight. Flasks were irradiated in triplicates with various doses, either with CONV-RT (8 Gy/min) or FLASH (average dose rate > 90 Gy/s, 3 Gy/pulse, instantaneous dose rate = 0.85 · 10^6^ Gy/s, total treatment time ≤ 170 ms) using a 10 MeV electron beam of a clinical linear accelerator (Elekta Precise, Elekta AB, Stockholm, Sweden)^26^. Doses were increased by 3 Gy/group, ranging from 0 to 21 Gy × 1. Dosimetry was verified using GafChromic EBT3 film (Ashland Advanced Materials, Bridgewater NJ). After irradiation, flasks were incubated in 37 °C for 7 days for colonies to form, and then stained using methylene-blue in ethanol for evaluation. Unirradiated control flasks were used to determine plating efficiency. Colonies containing at least 50 cells were defined as survivors.

### In vivo experiments

NS1 cells were prepared for inoculation by removal of the medium and washed gently with PBS. Trypsin (Invitrogen) was added and cells were incubated to detach the adherent cells. More medium was added and viable cells were counted. The cells were centrifuged at 1200 rpm for 5 min at 4 °C, and then the supernatant was removed. Afterwards the cell pellet was re-suspended in serum-free medium. 1000 cells/µl were used for intracranial injections and 500 cells/µl were used for subcutaneous injections.

Fischer 344 rats were used (Fischer Scientific, Germany). The rats were housed in pairs in rat cages with water and rat chow ad libitum. The animals were monitored daily, and those displaying signs of paresis, epilepsy, or declined general condition were euthanized in accordance to the ethical permission. All efforts were made to minimize animal suffering. Inoculations were performed in general anesthesia with isoflurane inhalation. All animals were randomized to their respective treatment groups at the initiation of the study.

In order to establish subcutaneous tumors, rats were inoculated with 50,000 NS1 cells subcutaneously day 0 in prone position and tumor site was carefully marked.

Animals who survived 91 days with no signs of tumor growth were re-challenged, with a tumor inoculation with NS1 cells on the contralateral side compared to the primary inoculation. The animals were followed until day 191 from the first inoculation, and were monitored regarding signs of tumor re-growth on either side.

In order to establish intracranial tumors, each rat received 5000 cells from the green fluorescent protein (GFP) tumor cell line, suspended in 5 µl of nutrient solution. Intracerebral tumor cell inoculation was done under isoflurane inhalation anaesthesia using a stereotactic frame and a 10 µl Hamilton syringe. The cells were injected on the right side of the cranium at a depth of 5 mm, 2 mm laterally from the sagittal suture, and 1 mm anterior to the coronal suture. The cranial burr hole was sealed with bone wax, and the incision was closed with absorbable suture.

### Radiotherapy

Prior to irradiation, the animals were anaesthetized by intraperitoneal injection of Ketalar/Rompun and positioned in custom-made PMMA boxes. Tumors were targeted using the crosshair of the linear accelerator light field (with the inoculation site as a reference point for intracranial tumors).

#### Subcutaneous tumors

Animals were irradiated in two fractions (day 8 and day 14), either with CONV-RT or FLASH, using the abovementioned 10 MeV electron beam. A circular radiation field with a diameter of 2 cm was used. CONV-RT 8 Gy × 2 was delivered at a source-to-surface distance (SSD) of 65 cm with an average dose rate of 8 Gy/min. For FLASH 8 Gy × 2, each fraction was delivered at SSD = 65 cm in 4 pulses with an average dose rate of 66 Gy/s, an instantaneous dose rate of 0.6 × 10^6^ Gy/s, and a total treatment time of 120 ms. Dosimetry was performed using GafChromic XD film (Ashland Advanced Materials, Bridgewater NJ) prior to each treatment. For FLASH delivery, a Farmer-type ionization chamber (NE 2505/3-3A) was used for relative output measurements during treatment.

#### Intracranial tumors

Animals were irradiated in two fractions (day 9 and day 13), either with CONV-RT 12.5 Gy × 2 or FLASH 12.5 Gy × 2. Both CONV-RT and FLASH were delivered at SSD = 67.5 cm using a field size of 1 × 1cm^2^. CONV-RT 12.5 Gy × 2 was delivered with an average dose rate of 8 Gy/min. For FLASH 12.5 Gy × 2, each fraction was delivered in 7 pulses with an average dose rate of 74 Gy/s, an instantaneous dose rate of 0.5 × 10^6^ Gy/s, and a total treatment time of 170 ms.

### Multiplex Assay

Luminex Multiplex Assay (Bio-Techne) was used on rat serum according to the manufacturer’s instructions, including the analytes GM-CSF, ICAM-1, IL-2, IL-6, IL-18, TIMP-1, TNF-alpha and VEGF.

### Gene analysis

In another set of control animals, inoculated as described above with either intracranial or subcutaneous NS1 cells, a part of the tumor was dissected and frozen in liquid nitrogen when the animals fulfilled criteria for euthanasia due to large tumor growth. RNA extraction was performed using RNeasy kit (Qiagen) according to the manufacturer’s instructions. RNA sequencing was performed at Center for Translational Genetics, Lund University. Only samples with adequate RNA quality could be assessed. Data was analyzed in R v.3.6.3 (R core team 2020).

#### Differential gene expression

Differential gene expressions between groups of samples (intracranial versus subcutaneous tumors) were assessed using edgeR v. 3.28.1^27–29^. All genes where two or more samples had fewer than 10 reads were removed prior to the analyses. The raw read counts were normalized to reads per million reads (rpmr) for each sample. Genes were considered differentially expressed if there were at least a twofold change between groups of samples and the fold change was significant (*p* < 0.05 after correction for multiple testing) after adjusting for expression levels using a generalized linear model provided in the R package.

### Statistics

SPSS was used for statistical evaluations, except the gene analysis, where R was used as described above. Normality was assessed using Shapiro–Wilk’s test and visual inspection of normality plots. Kruskal–Wallis and Mann–Whitney U-tests were performed for non-parametric data, and Bonferroni corrections were used in cases of multiple hypothesis testing. Log Rank Mantel-Cox was used to assess survival. *p* < 0.05 was used for significance.

## Data Availability

Results of gene datasets analyzed during the current study have been submitted to the Array Express repository (Accession Number E-MTAB-11674). Other data generated in the current study is available upon reasonable request, upon contact with the corresponding author.
